# Type I Interferon Regulation of HLA-F Expression in Human Trophoblasts During Viral Infection

**DOI:** 10.3390/v18060603

**Published:** 2026-05-26

**Authors:** Diana Manchorova, Jiahui Ding, Annie Thy Nguyen, Tanya Dimova, Sergey Slavov, Liubomir Djerov, Ruqun Zheng, Gil Mor

**Affiliations:** 1 C.S. Mott Center for Human Growth and Development, Department of Obstetrics and Gynecology, Wayne State University, Detroit, MI 48201, USA; diana_man4orova@abv.bg (D.M.); candy.ding@wayne.edu (J.D.); annie.nguyen@med.wayne.edu (A.T.N.); 2 Institute of Biology and Immunology of Reproduction “Acad. Kiril Bratanov”, Bulgarian Academy of Sciences, 1113 Sofia, Bulgaria; tanyadimova@yahoo.com; 3 Department of Biochemistry, Microbiology and Immunology, Wayne State University, Detroit, MI 48201, USA; 4 School of Medicine, Wayne State University, Detroit, MI 48201, USA; 5 Obstetrics and Gynecology Department, Medical University, University Obstetrics and Gynecology Hospital “Maichin Dom”, 1431 Sofia, Bulgaria; sergeislavov66@gmail.com (S.S.); bubodjerov@abv.bg (L.D.); 6 Department of Obstetrics and Gynecology, The Chinese University of Hong Kong, Hong Kong 999077, China; ruqunzheng@link.cuhk.edu.hk

**Keywords:** human leukocyte antigen F, type I interferon pathway, Zika virus, maternal–fetal interface

## Abstract

The role of human leukocyte antigen F (HLA-F) at the maternal–fetal interface (MFI) during viral infection and its regulation by interferon signaling remains poorly understood. Here, we investigated HLA-F expression and regulation in first-trimester trophoblast cells following activation of the type I interferon pathway and viral infection. We demonstrate that HLA-F is significantly upregulated at both mRNA and protein levels in response to Poly(I:C) and IFN-β in a dose- and time-dependent manner, suggesting its regulation as an interferon-stimulated gene (ISG). Zika virus (ZIKV) infection similarly induced HLA-F upregulation over time. In contrast, HSV-2 infection downregulated HLA-F mRNA while maintaining steady protein levels, indicative of virus-specific regulatory mechanisms. Moreover, we identified a soluble form of HLA-F secreted following Poly(I:C) stimulation. These findings reveal that HLA-F is dynamically regulated in trophoblasts during viral challenge and type I IFN signaling activation, supporting its broader immunomodulatory role in antiviral defense and immune tolerance at the MFI.

## 1. Introduction

Human pregnancy represents a unique immunological condition in which the maternal immune system must establish tolerance to a semi-allogeneic fetus while preserving effective antimicrobial immunity to protect both mother and fetus [1,2]. During implantation, the embryo must effectively communicate with maternal tissues while avoiding immune activation, thereby allowing proper placental development and the progression of pregnancy [3,4,5].

During the first trimester, the human placenta expands through the development of extravillous trophoblasts (EVTs), a specialized population of fetal cells that invade the maternal decidua and myometrium and transform uterine spiral arteries to facilitate increased blood flow and nutrient exchange required to support fetal development [6,7,8,9]. This complex immunological interaction relies heavily on crosstalk between EVTs and maternal immune cells, particularly uterine natural killer (NK) cells, macrophages, and T cells [10,11,12,13]. These interactions are largely mediated by ligands such as human leukocyte antigen (HLA) molecules and their receptors, including killer immunoglobulin-like receptors (KIRs), LILRB1/2, and natural killer cell receptors, which collectively promote immune tolerance at the maternal–fetal interface (MFI) [14,15]. EVTs express the polymorphic classical HLA-C molecule as well as the oligomorphic non-classical HLA-G, HLA-E, and HLA-F molecules [16].

Viral infections during pregnancy pose a significant threat to both maternal health and fetal development [17]. Zika virus (ZIKV) and herpes simplex virus type 2 (HSV-2) are among the most clinically significant viruses affecting pregnancy [18,19,20]. ZIKV is a positive-sense single-stranded RNA virus [21] that can cross the placental barrier and infect trophoblast cells, leading to severe outcomes such as fetal growth restriction and congenital Zika syndrome [22,23,24]. Type I interferon (IFN) signaling plays a critical role in restricting ZIKV replication, particularly during early pregnancy [25,26,27,28]. In contrast, HSV-2, a double-stranded DNA virus, establishes lifelong latency and has evolved several immune evasion strategies, including suppression of the interferon-β (IFN-β) signaling cascade [29].

Type I interferons, particularly interferon-β (IFN-β), play a central role in protecting trophoblasts and the developing placenta from viral infection [30]. Trophoblast cells exhibit a unique innate immune program characterized by constitutive or rapidly inducible production of IFN-β, which establishes an antiviral state both within infected cells and in neighboring cells through autocrine and paracrine signaling [28,31,32]. This signaling activates a broad network of interferon-stimulated genes (ISGs) that inhibit viral entry, replication, and spread at the maternal–fetal interface [33]. Previous work from our group demonstrated that trophoblast-derived IFN-β contributes to the remarkable resistance of placental cells to a wide range of viral pathogens, including both RNA and DNA viruses [28,32]. Furthermore, trophoblast-derived factors such as exosomes can transmit antiviral protection to surrounding maternal immune and epithelial cells [34,35]. Despite these findings, the downstream ISGs responsible for mediating this protective antiviral environment remain incompletely understood.

Human leukocyte antigen F (HLA-F) is a non-classical HLA class I molecule expressed at the maternal–fetal interface that interacts with several immune receptors, including ILT2, ILT4, and members of the KIR family [36,37,38], suggesting an important role in regulating immune responses during pregnancy. HLA-F can exist both as a peptide-bound complex and as an open conformer lacking β2-microglobulin [39], although the functional significance of these forms remains poorly understood. Reduced expression of HLA-F in extravillous trophoblasts has been associated with pathological conditions such as preeclampsia [40], and recent studies suggest that HLA-F may influence trophoblast metabolism and proliferation [41,42]. Given its capacity to engage immune regulatory receptors and modulate innate immune cell activity, HLA-F may contribute to shaping immune responses to viral infections at the maternal–fetal interface, where maintaining a balance between antiviral defense and immune tolerance is critical. However, despite evidence supporting the presence of HLA-F in placental tissues, the mechanisms regulating its expression and its role in antiviral immunity during pregnancy remain poorly defined. In particular, whether HLA-F is regulated by interferon signaling and contributes to antiviral responses at the maternal–fetal interface remains unknown.

In this study, we investigated the regulation and potential role of HLA-F at the maternal–fetal interface in the context of viral infection. We characterized the expression of HLA-F in first-trimester placental tissues and trophoblast cells and examined whether its expression is regulated by type I interferon signaling. Using IFN-β stimulation, the viral mimic poly(I:C), and infection with ZIKV and HSV-2, we assessed how antiviral pathways influence HLA-F expression. Our findings demonstrate that HLA-F is expressed in trophoblast cells of the placenta and is upregulated in response to IFN-β and viral stimuli, indicating that HLA-F is regulated as part of the trophoblast antiviral response. These results identify HLA-F as a previously underappreciated component of the immune regulatory network that contributes to antiviral defense at the maternal–fetal interface during early pregnancy.

## 2. Materials and Methods

### 2.1. Gestational Tissues Collection

Decidua and villi from first-trimester pregnancies were collected from 10 women undergoing legal termination of an intact healthy pregnancy (8–12 gestational weeks, gw). Third-trimester samples of decidua and villi were collected from 10 healthy women with a normal pregnancy (40 gw) following a spontaneous vaginal delivery or elective cesarean section. Informed consent for histological examinations of the harvested tissues was obtained from all patients in accordance with the ethical standard of the Helsinki declaration and approved by the Human Research Ethics Committee at the University Obstetrics and Gynecology Hospital “Maichin Dom” and the Medical University, Sofia, Bulgaria (No. 250528/20.04.22).

### 2.2. Histology and Immunohistochemistry (IHC)

The gestational tissues were fixed in HOPE^®^ (Polysciences, EuropeGmH, Hirschberg an der Bergstrasse, Germany), routinely processed, embedded in paraffin wax, and sectioned at 5–7 μm. Selected slides after routine hematoxylin and eosin (H&E) staining were subjected to IHC for in situ detection of HLA-F. A three-step biotin–streptavidin–HRP method and UltraTek HRP Anti-Polyvalent (DAB) Staining System (ScyTek, Logan, UT, USA) was used. Briefly, dewaxed and rehydrated sections were washed three times in PBS and incubated with Super Block (#AAA125, Sky Tek, Logan, UT, USA) to inhibit the non-specific binding. After PBS wash, the slides were subjected to endogenous peroxidase exhaustion by 3% H_2_O_2_ for 30 min at 37 °C. Then, the sections were incubated with primary polyclonal rabbit anti-human antibody against HLA-F (#14670-1-AP, Proteintech, Rosemont, IL, USA) or PBS (as a negative control) overnight at 4 °C in a humidified chamber. After PBS wash of the slides, the endogenous biotin was blocked (Biotin blocking kit, #BBK-IFU, Sky Tek), and then the washed sections were incubated with the biotinylated antibody for 10 min at room temperature (RT, ScyTek). The PBS-washed slides were then incubated with streptavidin–horseradish peroxidase for 10 min at RT (ScyTek, Logan, UT, USA). The color reaction was carried out with the chromogen—DAB (3,3 diaminobenzidine tetrahydrochloride). Nuclei were counterstained with Mayer’s hematoxylin and bluing reagent (Vector Laboratories, Inc., 6737 Mowry Ave Newark, CA 94560, USA). Finally, the sections were dehydrated, soaked in xylene, enclosed and examined by light microscopy. Sections from human tonsils processed in the same way served as positive controls for specificity of the HLA-F staining. Negative control staining, omitting biotinylated antibody or streptavidin-HRP complex, was included in each run.

### 2.3. Cell Culture and Infection

Immortalized human trophoblast Sw71 cells, confirmed to be Mycoplasma-free, were cultured in DMEM/F12 medium (REF: 11320-033, Gibco) containing 10% FBS, 10 mM HEPES (REF:15630-080), 0.1 mM MEM non-essential amino acids (REF: 11140-050), 1 mM sodium pyruvate (REF:11360-070), and 100 U/mL penicillin/streptomycin (REF:15140-122) (all from Gibco, Grand Island, NY, USA). The cells were cultured at 37 °C in a 5% CO_2_ incubator. For experiments involving treatment of Sw71 cells with synthetic viral RNA polyinosine–polycytidylic acid (Poly I:C) and human IFN-β, cells were seeded into 60 mm Petri dishes at a density of 2 × 10^5^ for 4, 8, 16, and 24 h incubations, or 1.75 × 10^5^ for 48 and 72 h incubations, in DMEM/F12 complete media supplemented with 10% FBS and cultured overnight. The following day, cells were pre-treated with DMEM/F12 containing 1% FBS for 4 h, after which they were exposed to the indicated concentrations of Poly I:C VacciGrade (10 mg, CAS number 31852-29-6, Invivogen, 10515 Vista Sorrento Pkwy, San Diego, CA 92121, USA) or human IFN-β recombinant protein (300-02BC-20UG, PeproTech, 5 Cedarbrook DrCranbury, NJ 08512, USA). The cell pellet and conditioned media were collected at the specified time points. For Zika viral infection studies, cells were seeded into 60 mm Petri dishes at a density of 2 × 10^5^ cells for a 24 h incubation or 1.75 × 10^5^ cells for 48 and 72 h incubations. For HSV, 1.75 × 10^5^ cells were seeded for 24 or 48 h. The following day, cells were pre-treated as described and infected with either ZIKV or HSV at the indicated MOI. After a 1 h incubation with gentle agitation every 20 min, the viral inoculum was removed, cells were washed with phosphate-buffered saline (PBS, REF: 10010-023, Gibco), and fresh complete DMEM/F12 medium containing 10% FBS was added. Cells were maintained in this medium for the remainder of the experiment. At designated time points post-infection, cell pellets were collected for analysis.

### 2.4. Viruses

Zika virus strain FSS 13025, initially isolated in Cambodia in 2010, was sourced from the World Reference Center for Emerging Viruses and Arboviruses at the University of Texas Medical Branch in Galveston, as previously reported [43]. The virus was propagated by infecting monolayers of Vero cells (African green monkey kidney cells) with the viral stock. Once widespread cytopathic effects were observed, the supernatant containing the virus was collected and centrifuged. The resulting virus stocks were divided into aliquots and stored at −80 °C. Viral titers were measured using a plaque assay. HSV-2 was obtained from ATCC (catalog #VR-540; Manassas, VA, USA), expanded by passaging in Vero cells, and its concentration was assessed via plaque assay.

### 2.5. RNA Extraction and Quantitative PCR

Total RNA was isolated using the RNeasy Mini Kit (Qiagen, Cat. No. 74106, 19300 Germantown RoadGermantown, MD 20874, USA) following the manufacturer’s instructions. The final RNA pellet was resuspended in 30 µL of RNase-free water. RNA integrity and concentration were evaluated by spectrophotometry (BioTek Epoch Microplate Spectrophotometer, 1000 Alfred Nobel Drive Hercules, CA 94547, USA), and only samples with 260/280 ratios ≥1.8 were used for downstream analysis. For cDNA synthesis, 1 µg of total RNA per sample was reverse transcribed using the iScript cDNA Synthesis Kit (Cat. No. 1708891) with a Bio-Rad DNA Engine Thermal Cycler. Quantitative real-time PCR was performed using iTaq Universal SYBR Green Supermix (REF: 1725124, Bio-Rad) and gene-specific primers for HLA-F (forward: GCTGCTGTGATGTGGAGGAAGA; reverse: GTATGTTCGTGAGGCACAAGTGC) and GAPDH (forward: GTCTCCTCTGACTTCAACAGCG; reverse: ACCACCCTGTTGCTGTAGCCAA). Reactions were performed on a CFX96 Real-Time PCR Detection System (Bio-Rad, Hercules, CA, USA). Samples were run in technical duplicates and normalized to GAPDH. Relative gene expression was calculated using the 2^−ΔΔCt^ method [44].

### 2.6. Quantification of Viral Transcript by qRT–PCR

Zika viral transcripts were determined using one-step quantitative reverse transcription PCR (qRT–PCR). Viral RNA was extracted from Zika virus stock using the QIAamp MinElute Virus Spin Kit (Qiagen, Cat. No. 52906) and used to generate a standard curve through 10-fold serial dilutions. Total RNA (1 µg) from experimental samples was analyzed using the GoTaq Probe 1-Step RT-qPCR System (Promega, Cat. No. A6120, Promega Corporation, 2800 Woods Hollow RoadMadison, WI 53711, USA) on a CFX96 Real-Time PCR System (Bio-Rad). Zika virus detection was carried out using forward 5′-CCGCTGCCCAACACAAG-3′ and reverse 5′-CCACTAACGTTCTTTTGCAGACAT-3′, along with a FAM-labeled hydrolysis probe: 5′-AGCCTACCTTGACAAGCAGTCAGACACTCAA-3′ (ZEN/IBFQ) [45]. Thermocycling conditions included reverse transcription at 45 °C for 15 min, initial denaturation at 95 °C for 2 min, followed by 40 cycles of 95 °C for 15 s and 60 °C for 1 min. Viral RNA levels were quantified by comparing the Ct values of samples to the standard curve. qPCR amplification conditions for HSV-2 viral transcript included an initial denaturation at 95 °C for 10 min, followed by 40 cycles of 95 °C for 10 s, 66 °C for 5 s, and 72 °C for 6 s. A melt curve analysis was performed at the end with a 65 °C start, increasing to 95 °C in 0.1 °C increments. Viral RNA levels were quantified by qRT-PCR using HSV-2 glycoprotein B (gB) primers (Forward: AGACCAGGGCCGCTGATC; reverse: GCGCTGGACCTCCGTGTAG) by comparing Cq values between non-infected and infected samples.

### 2.7. Protein Isolation and Western Blotting

Cells were lysed on ice for 20 min using Cell Lysis Buffer (10X) (REF:9803S, Cell Signaling Technology, 3 Trask Lane, Danvers, MA 01923, USA) supplemented with protease inhibitor cocktail (Roche Diagnostics, REF: 11767900, Roche Diagnostics Corporation, 9115 Hague RoadIndianapolis, IN 46256, USA) and PMSF Phenylmethylsulfonyl fluoride (REF: 36978, Thermo Scientific, 168 Third Avenue, Waltham, MA 02451, USA). Lysates were centrifuged at 13,000× *g* for 15 min at 4 °C. Protein concentrations were measured using the Pierce BCA Protein Assay Reagent A and reagent B (Thermo Fisher Scientific, 168 Third Avenue Waltham, MA 02451, USA (A) Cat. No. 23228 and (B) REF:1859078). Equal amounts of protein (20 µg per sample) were separated on 12% SDS–polyacrylamide gels and transferred to PVDF membranes (Millipore, 400 Summit Drive Burlington, MA 01803, USA). Membranes were blocked with 5% nonfat dry milk (REF: DSM17200, DOT Scientific, 4165 Lippincott Blvd, Burton, MI 48519, USA) in PBS containing 0.05% Tween-20 (PBS-T) for 1 h at room temperature. After blocking, membranes were incubated overnight at 4 °C with primary antibodies diluted in 1% milk in PBS-T. The following day, membranes were washed and incubated with appropriate HRP-conjugated secondary antibodies (diluted in 1% milk in PBS-T) for 1 h at room temperature. Detection was performed using Clarity Western ECL Substrate (REF:1705061, Bio Rad), and images were acquired using Luminescent image analyzer (Model Image Quant LAS 500, 75184 Uppsala, Sweden). Primary antibodies were used at the following dilutions: rabbit anti-HLA-F (1:2000; Cat. No 14670-1-AP, Proteintech), mouse anti-GAPDH (1:10,000; Cat. No 60004-1-Ig, Proteintech). Secondary antibodies (goat anti-rabbit, REF: PI-1000 or horse anti-mouse, REF: PI-2000, both from Vector Laboratories Vector Laboratories, Inc., 6737 Mowry AvenueNewark, CA 94560, USA) were used at a 1:10,000 dilution in blocking buffer.

### 2.8. Conditioned Media Collection and Concentration

Conditioned media (CM) were collected from each experimental group and initially centrifuged at 1500 rpm for 5 min at room temperature (RT) to remove cellular debris and dead cells. For each group, two CM samples were pooled, resulting in a total volume of approximately 3–3.5 mL. The pooled CM was then concentrated using Amicon Ultra-0.5 centrifugal filters with a 30 kDa molecular weight cutoff (REF: UFC803096, Millipore). Samples were centrifuged at 4000× *g* for 15 min at RT. The resulting concentrated CM was carefully collected, and the total volume was measured. Subsequently, 20 μL of each concentrated CM sample was used for WB analysis.

### 2.9. Immunofluorescent Staining

Sw71 cells were cultured for 24–48 h in sterile chamber slides (Falcon, Cat. No. 354108, Corning, NY, USA) placed on glass slides. Following incubation, cells were fixed in 4% paraformaldehyde (PFA, REF: J19943-K2, Thermo Scientific) for 1 h at RT. After washing with PBS, non-specific binding was blocked by incubating the cells with 10% goat serum (Cat. No. 31873, Thermo Fisher Scientific) for 30 min at RT. Cells were then incubated with rabbit anti-human polyclonal HLA-F antibody (Proteintech, Cat. No. 14670-1-AP), followed by a secondary antibody: Alexa Fluor 555-conjugated goat anti-rabbit IgG (H + L) (Invitrogen, Cat. No. A-21428, 2 mg/mL, Waltham, MA USA), used at a working concentration of 4 µg/mL. Antibody validation was based on the manufacturer’s reported validation for the immunofluorescent staining, together with appropriate negative controls, including secondary-only controls, to assess nonspecific staining. Slides were imaged using a Nikon Eclipse E90i epifluorescence microscope (model D-PS, Tokyo, Japan), with filter sets for Texas Red and DAPI channels.

### 2.10. FACS Staining and Flow Cytometry Analysis

Flow cytometry was performed to evaluate the surface and intracellular expression of HLA-F in the Sw71 cell line. Cells were counted and adjusted to 1 × 10^6^ per sample and transferred to FACS tubes for staining. Samples were processed in two parallel staining protocols: one for surface (non-permeabilized) staining and another for intracellular (permeabilized) detection. For extracellular staining, cells were first incubated with anti-HLA-F antibody (CoraLite^®^ Plus 488, Cat. No. CL488-66819 Proteintech) for 30 min at 4 °C in the dark. After washing with ice-cold FACS buffer (PBS with 1% BSA), apoptosis was assessed using the Annexin V–PE-Cy7 (BioLegend, 8999 BioLegend Way, San Diego, CA 92121, USA, Cat. No. 640950). Briefly, cells were centrifuged, resuspended in 200 µL of Annexin V binding buffer (Cat. No.: 422201, BioLegend), and incubated with 10 µL of Annexin V for 10 min at room temperature in the dark. After staining, the samples were washed with Annexin V binding buffer and fixed in 4% paraformaldehyde (PFA) for 15 min and washed in FACS buffer. For intracellular staining, cells were first stained with Annexin V as described above, followed by fixation for 40 min at room temperature. Cells were then permeabilized using 1× permeabilization buffer (Thermo Fisher Scientific) for 40 min, washed with permeabilization buffer, and stained with anti-HLA-F antibody for 1 h at room temperature. After washing with permeabilization buffer, cells were fixed again in 4% PFA and washed in FACS buffer. Unstained and single-stained controls were included to set gates and perform compensation. Cell clusters were excluded using single-cell gating. Samples were acquired using a CytoFLEX Flow Cytometer (Beckman Coulter Life Sciences, 5350 Lakeview Parkway S Drive, Indianapolis, IN 46268, USA), and data analysis was conducted in FlowJo software v10 (Treestar, San Carlos, CA, USA).

### 2.11. Statistical Analysis

All statistical analyses were performed using GraphPad Prism version 9 (GraphPad Software, San Diego, CA, USA). Data are presented as mean ± standard error of the mean (SEM). Comparisons between two groups were conducted using an unpaired Student’s *t*-test. For comparisons among multiple groups, one-way ANOVA was used. When data did not meet the assumptions of normality, appropriate nonparametric tests were applied. A *p* value < 0.05 was considered statistically significant.

## 3. Results

### 3.1. Expression of HLA-F in the Placenta During Normal Pregnancy (Early and Term)

Our first objective was to determine whether HLA-F protein is expressed at the maternal–fetal interface during pregnancy and to define its cellular localization. To address this question, we performed immunohistochemical staining of placental and decidual tissues using a specific anti-HLA-F antibody. Placental samples were obtained from first-trimester elective terminations (8 gestational weeks) and from third-trimester placentas collected after normal vaginal delivery (40 gestational weeks). None of the analyzed samples had clinical or pathological evidence of infection.

**Figure 1:**
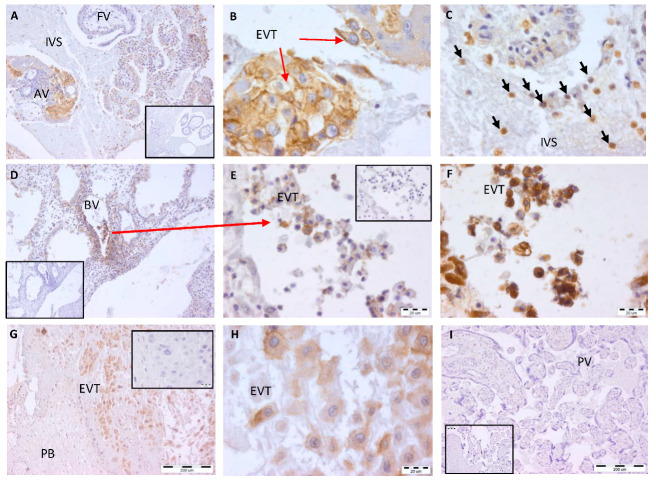
Expression of human leukocyte antigen F (HLA-F) in decidual and placental villous tissues during healthy early and term pregnancy. (**A**) Invading EVT from the anchoring placental villa strongly expressed HLA-F; floating placental villi are HLA-F-negative (8 gw). (**B**) The same is shown at higher magnification, EVTs (red arrows) strongly express HLA-F on their surface (8 gw). (**C**) HLA-F-positive immune cells (black arrows) into the intervillous space (8 gw). (**D**) Decidua with blood vessels (12 gw). (**E**) Higher magnification of D, a group of intravascular EVT expressing HLA-F (red arrow) (12 gw). (**F**) The same slide stained for HLA-G, note the relatively strong HLA-G signal by the intravascular EVT (12 gw). (**G**) Placental bed contains numerous HLA-F-positive EVT (40 gw). (**H**) The same is shown at higher magnification, note the cytoplasmic HLA-F staining of the EVT (40 gw). (**I**) HLA-F-negative placental villi of a term pregnancy placenta (40 gw). Representative images of n = 10 for each group. EVT—extravillous trophoblast cell; IVS—intervillous space; AV—anchoring placental villa; FV—floating placental villa; BV—blood vessel; PB—placental bed; PV—placental villa. Magnification: 100× objective (**B**,**C**,**E**,**F**,**H**), 40× objective (**A**,**G**), 20× objective (**D**,**I**). The negative controls are given as inserts.

Analysis of first-trimester placental samples revealed positive immunoreactivity for HLA-F in extravillous trophoblasts (EVTs) located within the anchoring villi (Figure 1A). In contrast, syncytiotrophoblasts, cytotrophoblasts, and the mesenchymal core of floating villi were negative for HLA-F expression (Figure 1A, inner square). Examination at higher magnification demonstrated strong HLA-F staining along the EVT membrane (Figure 1B, red arrows), together with detectable cytoplasmic staining (Figure 1B). In addition, individual immune cells located within the intervillous space (IVS) displayed positive HLA-F staining (Figure 1C, black arrows).

Further analysis of decidual samples revealed positive HLA-F staining in intravascular EVTs located adjacent to decidual blood vessels (Figure 1D,E). Higher magnification confirmed HLA-F expression in these invading EVTs (Figure 1E). To verify that these HLA-F-positive cells corresponded to EVTs, we performed staining for HLA-G, a well-established EVT-specific marker. Staining of the same tissue sections for HLA-G confirmed the identity of these cells, showing robust HLA-G expression in the same cell population (Figure 1F). These findings indicate that HLA-F expression during early pregnancy is primarily associated with EVTs.

We next evaluated HLA-F expression in term placentas (40 gestational weeks). Similar to the pattern observed in first-trimester samples, HLA-F-positive cells were detected within the EVT population in the placental bed (Figure 1G), while decidual cells remained negative (Figure 1G, inner square). Examination of intracellular localization revealed that HLA-F staining in term EVTs was predominantly cytoplasmic (Figure 1H). Consistent with observations in early pregnancy, placental villi were negative for HLA-F expression (Figure 1I).

Together, these findings demonstrate that HLA-F is expressed in human placental tissue and is primarily localized to extravillous trophoblasts at both early and late stages of pregnancy.

### 3.2. Poly(I:C) Induced Expression of HLA-F in Sw71 Trophoblast Cells

**Figure 2:**
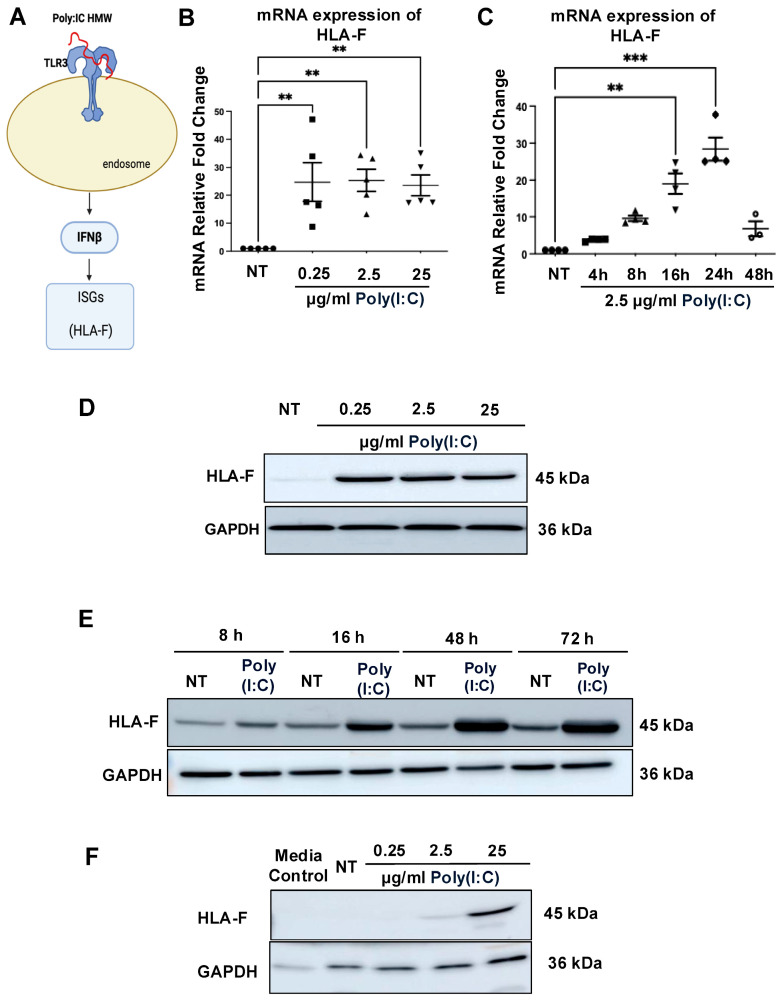
Poly(I:C) induces HLA-F mRNA and protein expression in a dose- and time-dependent manner in Sw71 trophoblast cells. Sw71 cells were treated with high-molecular-weight Poly(I:C) to evaluate HLA-F expression at both mRNA and protein levels. (**A**) Diagram illustrating the interferon-β signaling pathway, which is activated by viral mimics such as Poly(I:C). (**B**) Sw71 cells were treated with increasing doses of Poly(I:C) (0.25, 2.5, and 25 µg/mL) for 24 h, and total RNA was collected to measure HLA-F mRNA expression by qRT–PCR. ** *p* < 0.01 by one-way ANOVA (n = 5). (**C**) Sw71 cells were treated with 2.5 µg/mL Poly(I:C) for 4–48 h, and HLA-F mRNA levels were analyzed by qRT–PCR. The data did not follow a normal distribution, and a nonparametric one-way ANOVA was used. ** *p* < 0.01 and *** *p* < 0.001 (n = 3–4). (**D**) Protein was collected from Sw71 cells treated with increasing doses of Poly(I:C) (0.25, 2.5, and 25 µg/mL) for 24 h, and HLA-F protein levels were assessed by Western blot. GAPDH served as a loading control. (**E**) Time-course analysis of HLA-F protein expression after treatment with 2.5 µg/mL Poly(I:C) for 8–72 h, evaluated by Western blot. GAPDH served as a loading control. NT, no treatment group. (**F**) Sw71 cells were treated with increasing doses of Poly(I:C) (0.25, 2.5, and 25 µg/mL) for 24 h, and concentrated CM were collected for Western blot analysis. Soluble HLA-F was detected in a dose-dependent manner, with the strongest signal at 25 µg/mL.

Our next objective was to characterize the expression and regulation of HLA-F in trophoblast cells. Activation of TLR3 by double-stranded RNA (dsRNA) in trophoblasts triggers a well-characterized antiviral signaling cascade involving the adaptor protein TRIF, phosphorylation of IRF3, and subsequent induction of IFN-β and interferon-stimulated genes (ISGs) [46] (Figure 2A). Based on this pathway, we investigated whether HLA-F is an inducible ISG involved in this antiviral response. To address this question, we used the well-characterized human first-trimester trophoblast cell line Swan71 (Sw71), previously described in multiple studies [32,47]. Cells were stimulated with high-molecular-weight polyinosinic–polycytidylic acid (Poly(I:C)), a synthetic analog of viral double-stranded RNA and a ligand for TLR3 [47,48]. Sw71 cells were treated with increasing concentrations of Poly(I:C) (0.25, 2.5, and 25 µg/mL) for 24 h, and HLA-F mRNA expression was measured by qPCR. Poly(I:C) stimulation resulted in a significant increase in HLA-F mRNA levels, which was detectable even at the lowest dose tested (Figure 2B).

To further define the kinetics of this response, we performed a time-course experiment using 2.5 µg/mL Poly(I:C) and collected cells at 4, 8, 16, 24, and 48 h post-treatment. HLA-F mRNA expression was detected as early as 4 h, peaked at approximately 16 h, and declined by 48 h, indicating a rapid but transient transcriptional response to dsRNA stimulation (Figure 2C).

We next determined whether the observed transcriptional changes were reflected at the protein level. Consistent with the mRNA results, Poly(I:C) treatment induced a dose-dependent increase in HLA-F protein expression (Figure 2D). Similarly, time-course analysis revealed increased HLA-F protein levels following Poly(I:C) stimulation, with detectable expression beginning at approximately 16 h and further increasing at 48 and 72 h post-treatment (Figure 2E).

HLA-G, a member of the non-classical MHC class I molecules, exists in both membrane-bound and soluble forms [48]. Because we observed cytoplasmic staining in EVTs, we questioned whether HLA-F, in addition to a membrane-associated form, might also exist as a secreted protein. To test this possibility, we collected conditioned media (CM) from trophoblast cells treated with increasing doses of Poly(I:C) (0.25, 2.5, and 25 µg/mL) or vehicle control and analyzed the samples by Western blot. Notably, while control cells did not show detectable levels of secreted HLA-F, increased levels of soluble HLA-F were detected in the CM of Poly(I:C)-treated cells, particularly at the highest dose (25 µg/mL) (Figure 2F). Together, these findings demonstrate that HLA-F expression in trophoblast cells is inducible by viral dsRNA signaling and support the possibility that soluble HLA-F participates in antiviral immune responses at the maternal–fetal interface.

### 3.3. IFN-β Treatment Led to a Dose- and Time-Dependent Increase in HLA-F Expression in Sw71 Trophoblast Cells

Poly(I:C) is a strong inducer of type I IFN-β expression [49,50]. Based on this observation, we hypothesized that the increase in HLA-F expression observed following Poly(I:C) treatment may be mediated by IFN-β. To test this hypothesis, Sw71 cells were treated with increasing concentrations of IFN-β at different time points, and HLA-F expression was evaluated at both the mRNA and protein levels using qRT-PCR and Western blot, respectively.

**Figure 3:**
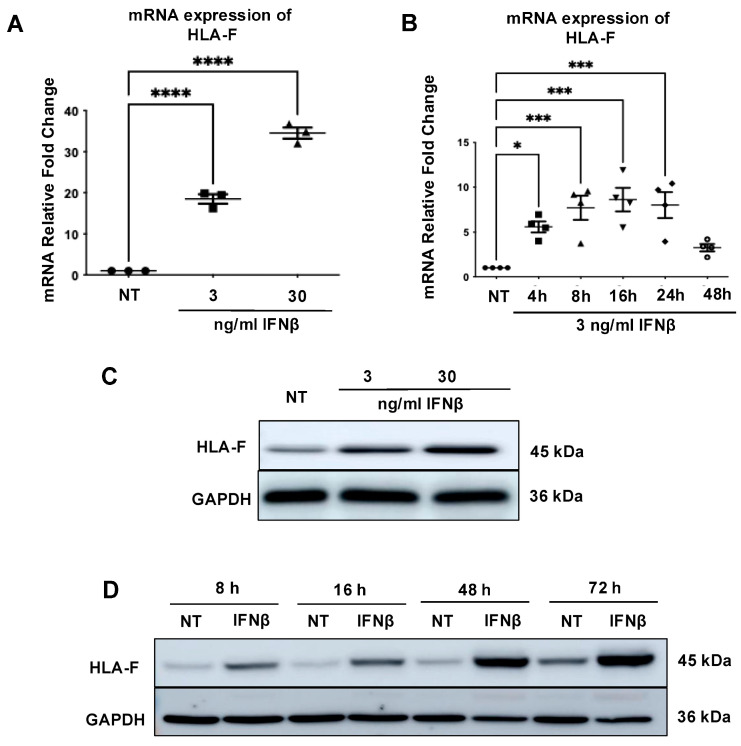
IFN-β induces HLA-F mRNA and protein expression in a dose- and time-dependent manner in Sw71 trophoblast cells. (**A**) Sw71 cells were treated with increasing doses of IFN-β (3 and 30 ng/mL) for 8 h, and RNA was collected to assess HLA-F mRNA expression by qRT–PCR. **** *p* < 0.0001 by one-way ANOVA (n = 3). (**B**) Sw71 cells were treated with 3 ng/mL IFN-β for 4–48 h, and HLA-F mRNA expression was measured by qRT–PCR. * *p* < 0.05 and *** *p* < 0.001 by one-way ANOVA (n = 4). (**C**) Protein was collected from Sw71 cells treated with 3 and 30 ng/mL IFN-β for 24 h, and HLA-F protein expression was analyzed by Western blot. GAPDH served as a loading control. (**D**) Sw71 cells were treated with 3 ng/mL IFN-β for 8–72 h, and HLA-F protein levels were assessed by Western blot. GAPDH served as a loading control.

Both concentrations of IFN-β (3 ng/mL and 30 ng/mL) significantly increased HLA-F mRNA levels after 24 h of treatment (Figure 3A). To further characterize the kinetics of this response, we performed a time-course experiment using 3 ng/mL IFN-β and collected cells at 4, 8, 16, 24, and 48 h post-treatment. HLA-F mRNA expression increased as early as 4 h, peaked at 16 h, and began to decline by 48 h (Figure 3B).

Next, we examined whether the increase in HLA-F mRNA expression was reflected at the protein level (Figure 3C,D). Treatment with IFN-β resulted in a dose-dependent increase in HLA-F protein expression following exposure to both 3 ng/mL and 30 ng/mL IFN-β (Figure 3C). In the time-course experiment, HLA-F protein expression was detectable at 8 h post-treatment and continued to increase, remaining elevated through 72 h (Figure 3D). These results support the notion that the induction of HLA-F observed following Poly(I:C) stimulation is mediated, at least in part, through IFN-β signaling.

### 3.4. Zika Virus Infection Induces HLA-F Expression in Trophoblast Sw71 Cells

Similarly to Poly(I:C), Zika virus (ZIKV), a positive-sense single-stranded RNA virus, activates TLR3 signaling and induces type I interferon responses [51]. Because ZIKV can infect placental cells [43] and has been associated with adverse pregnancy outcomes [52], we next investigated whether viral infection regulates HLA-F expression in trophoblast cells. Sw71 trophoblast cells were infected with the Cambodia strain of ZIKV (MOI = 0.0004) for 1 h, followed by replacement with fresh culture media. Viral replication and HLA-F expression were subsequently evaluated. These MOIs do not induce cell death and therefore are a good model to evaluate the anti-viral response.

**Figure 4:**
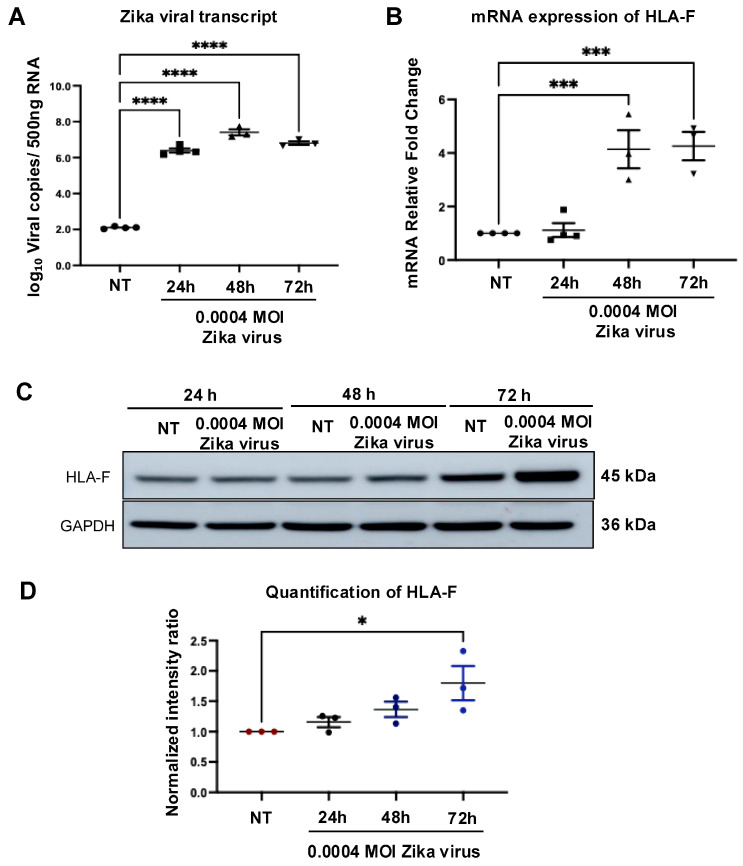
Expression of HLA-F in Sw71 trophoblast cells following Zika virus infection. Sw71 cells were infected with ZIKV (Cambodia strain, MOI = 0.0004) for 1 h and cultured for up to 72 h to assess HLA-F expression at the RNA and protein levels. (**A**) ZIKV viral gene expression measured by qRT–PCR showed a time-dependent increase, confirming productive infection. **** *p* < 0.0001 by one-way ANOVA (n = 3–4). (**B**) HLA-F mRNA expression was measured by qRT–PCR at 24, 48, and 72 h post-infection. *** *p* < 0.001 by one-way ANOVA (n = 3–4). (**C**) Protein expression of HLA-F was evaluated by Western blot at 24, 48, and 72 h post-infection. GAPDH served as a loading control. (**D**) Quantification of Western blot data from three independent experiments showed a significant increase in HLA-F protein expression at 72 h post-infection. * *p* < 0.05 by one-way ANOVA.

Throughout the course of infection, Sw71 cells exhibited no morphological signs of apoptosis or significant changes in growth compared with uninfected controls. Viral RNA levels measured by qRT–PCR confirmed productive infection, with detectable viral RNA at 24 h post-infection, increased levels at 48 h, and a slight decline at 72 h (Figure 4A).

ZIKV infection induced upregulation of HLA-F mRNA expression in Sw71 trophoblast cells, with increased levels observed at 48 h post-infection and further elevation at 72 h (Figure 4B). Consistent with these transcriptional changes, HLA-F protein levels showed a modest increase at 48 h and a more pronounced increase at 72 h post-infection (Figure 4C), indicating that the increase in mRNA expression translated into increased protein production. Quantification of Western blot data from three independent experiments confirmed a statistically significant increase in HLA-F protein expression at 72 h post-infection (Figure 4D).

These findings are consistent with our observation that IFN-β signaling induces HLA-F expression, suggesting that viral sensing pathways activated during ZIKV infection may drive HLA-F upregulation.

### 3.5. Lack of HLA-F Induction by HSV-2 Suggests Dependence on Type I IFN Activation

**Figure 5:**
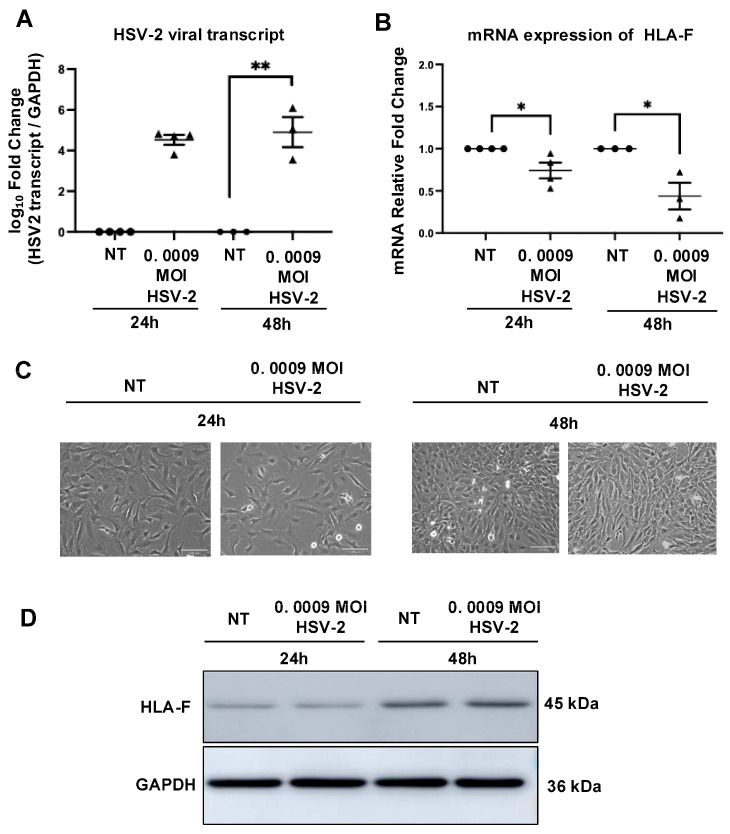
Downregulation of HLA-F expression in Sw71 trophoblast cells following HSV-2 infection. (**A**) HSV-2 viral gene expressions were quantified by qRT-PCR at 24 and 48 h post-infection. Viral transcript levels were increased at 24 h (non-significant, Wilcoxon signed-rank test for not normally distributed data), and a significant increase was seen at 48 h (** *p* < 0.01, unpaired Student’s *t*-test), confirming productive infection. (**B**) Sw71 cells were infected with HSV-2 for 24 and 48 h, and HLA-F mRNA expression was analyzed by RT-qPCR. A significant decrease in expression was observed at both time points compared to untreated controls. * *p* < 0.05 by unpaired Student *t*-test. (**C**) Transmitted light mode images of Sw71 cells at 24 and 48 h post-infection revealed no visible morphological alterations or Infection-induced cell damage compared to controls. (**D**) Western blot analysis of HLA-F protein levels showed no induction following HSV-2 infection at either time point. GAPDH was used as a loading control.

HSV-2 is a double-stranded DNA virus known to suppress the Type I interferon response [53] and is a well-recognized cause of severe pregnancy complications, including miscarriage, preterm birth, and neonatal infection [54,55,56]. To investigate whether the upregulation of HLA-F observed during ZIKV infection depends on Type I interferon signaling, Sw71 trophoblast cells were infected with HSV-2 (MOI = 0.0009) for 1 h, followed by replacement with fresh culture medium. Infection efficiency was evaluated by qRT-PCR at 24 and 48 h post-infection. HSV-2 viral transcripts were detectable at 24 h and significantly increased by 48 h, confirming productive infection and viral replication in Sw71 trophoblast cells (Figure 5A).

We next examined HLA-F mRNA expression in HSV-2-infected cells by RT-qPCR. In contrast to the upregulation observed following ZIKV infection, HSV-2 infection resulted in a significant downregulation of HLA-F expression at both 24 and 48 h post-infection (Figure 5B).

To determine whether HSV-2 infection induced cytopathic effects, Sw71 cells were monitored by light microscopy prior to collection at 24 and 48 h post-infection. At this viral concentration, no visible signs of apoptosis or alterations in cell morphology or growth were observed compared with uninfected controls, indicating that HSV-2 infection at this dose and time did not induce detectable cytopathic effects (Figure 5C) and therefore the decrease in HLA-F expression is not due to cell death but a response to HSV2.

Consistent with the reduction in HLA-F mRNA levels, no increase in HLA-F protein expression was detected in HSV-2-infected Sw71 cells (Figure 5D). Although viral infections can often induce HLA gene expression as part of a general cellular stress response [57,58,59,60], these findings indicate that HLA-F induction is not a universal consequence of viral infection. Instead, its upregulation appears to be specifically associated with RNA viruses such as ZIKV that trigger a strong Type I interferon response.

### 3.6. Intracellular Expression of HLA-F Is Associated with the Golgi Apparatus

**Figure 6:**
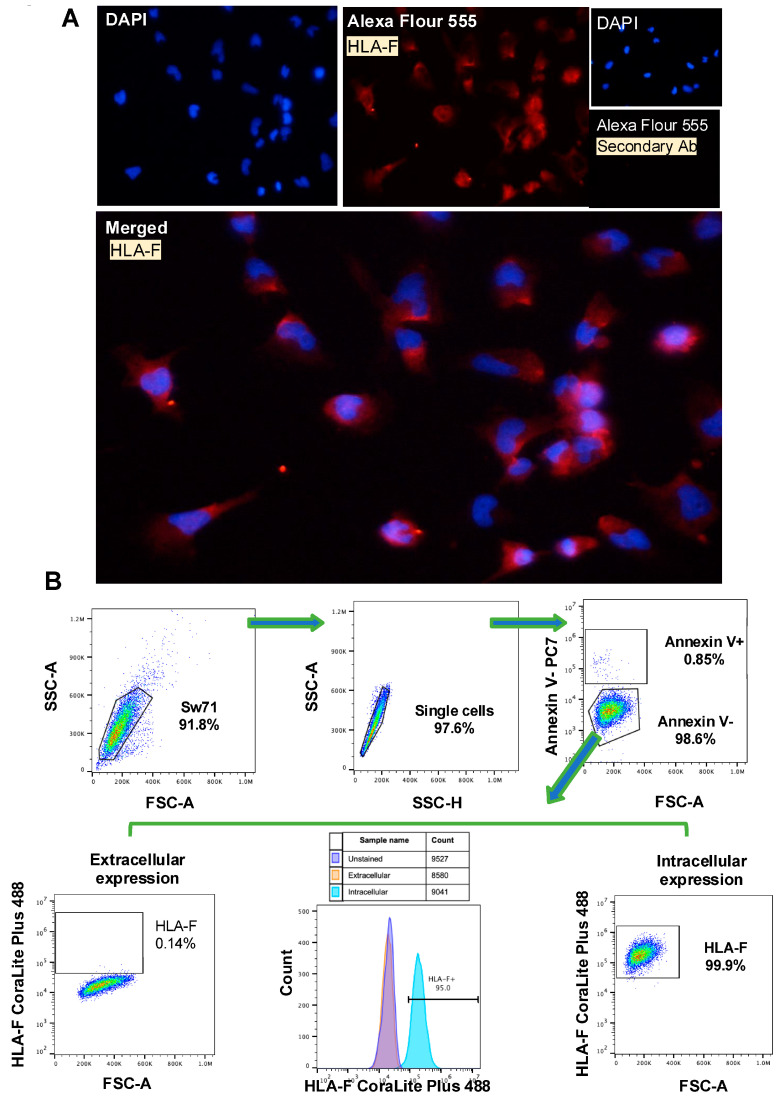
Expression of HLA-F in Sw71 trophoblast cells assessed by immunofluorescence and flow cytometry. (**A**) Sw71 cells were cultured in glass chambers and stained for HLA-F using immunocytochemistry. HLA-F (red, Alexa Fluor 555) was predominantly localized intracellularly in a perinuclear pattern, consistent with ER/Golgi localization. Nuclei were counterstained with DAPI (blue). Insets show negative controls without primary antibody, confirming staining specificity. (**B**) Flow cytometry for extracellular or intracellular HLA-F expression in Sw71 cells. Representative plots showing the gating strategy for flow cytometry. Sw71 cells were gated based on size, singlets, and viability (Annexin V-negative). Flow cytometry analysis of HLA-F expression in viable Sw71 cells. Surface (**left**), intracellular (**right**), and overlay histogram (**middle**). Intracellular HLA-F was detected in nearly all cells, while surface staining overlapped with the unstained control, indicating no surface expression.

Having confirmed that HLA-F is expressed in Sw71 trophoblast cells and is responsive to various external stimuli, we next investigated its subcellular localization at the basal level without any stimulation. Consistent with the localization observed in placental samples (Figure 1), immunofluorescence (IF) analysis using an anti-HLA-F antibody revealed that HLA-F in Sw71 cells exhibited a predominantly intracellular, perinuclear distribution. This pattern is consistent with a potential association with organelles involved in protein processing and trafficking, such as the endoplasmic reticulum or Golgi apparatus (Figure 6A). Additional organelle-specific makers will be required to determine the precise subcellular compartment associated with HLA-F localization.

To further validate these findings, we performed flow cytometry (FACS) analysis to assess both surface and intracellular levels of HLA-F (Figure 6B). The gating strategy included identification of Sw71 cells, selection of single cells, and exclusion of apoptotic cells by gating out Annexin V-positive populations (Figure 6B). Within this viable population, HLA-F expression was evaluated separately at the cell surface (left) and intracellularly (right), allowing a more detailed characterization of its localization (Figure 6B).

The results showed that nearly all Sw71 cells were positive for intracellular HLA-F, while surface expression was undetectable (Figure 6B). Overlayed histograms demonstrated that surface HLA-F expression was indistinguishable from the unstained control, indicating an absence of detectable surface expression. In contrast, intracellular staining produced a clear rightward shift, confirming the presence of HLA-F within the cells (Figure 6B). This intracellular localization is consistent with our observation that HLA-F can be detected in conditioned media following Poly(I:C) stimulation, suggesting that intracellular pools may contribute to its secretion.

## 4. Discussion

**Figure 7:**
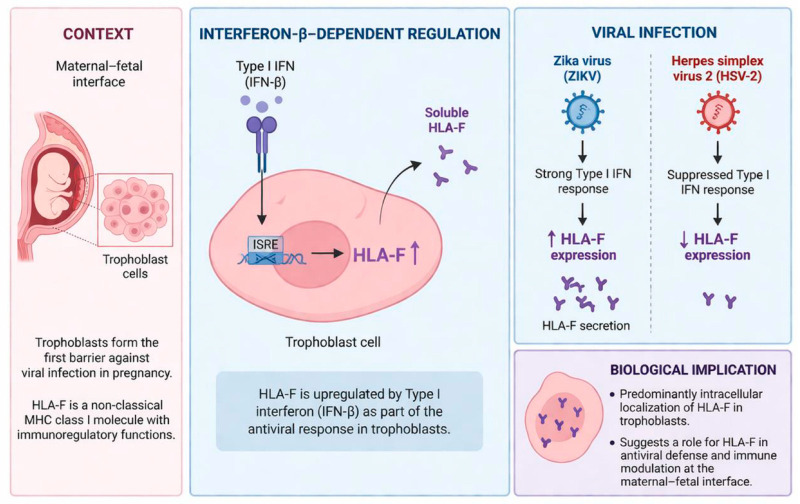
Interferon-β-dependent regulation of HLA-F in human trophoblasts during viral infection. HLA-F is an interferon-stimulated molecule in trophoblasts that is induced by antiviral responses and may contribute to immune regulation at the maternal–fetal interface.

We report here the first characterization of HLA-F expression and regulation in the first-trimester human placenta and in trophoblast Sw71 cells. Our findings identify type I interferon signaling, particularly IFN-β, as a key regulator of HLA-F expression in response to viral stimuli. Activation of antiviral pathways using poly(I:C) not only induced HLA-F expression but also led to the detection of secreted HLA-F, suggesting that this molecule may exert paracrine or endocrine immunomodulatory functions at the maternal–fetal interface (MFI). In contrast, infection with HSV-2, a virus known to suppress type I interferon responses as a strategy to evade the host’s innate immune response [28,61], resulted in marked inhibition of HLA-F expression, further supporting a functional link between interferon signaling and HLA-F regulation. Notably, in addition to the HLA-F membranal expression observed in EVTs, we also observed HLA-F expression localized intracellularly in trophoblasts, and a secreted form detectable in the supernatant of trophoblast cells upon stimulation. Together, these results suggest that HLA-F operates as an interferon-immunoregulatory molecule in trophoblasts, linking antiviral sensing to immune adaptation at the maternal–fetal interface (Figure 7).

HLA-F is a non-classical MHC class I molecule belonging to the HLA-Ib family, which also includes HLA-E and HLA-G [62,63]. In contrast to classical MHC class I molecules, which primarily function in antigen presentation to CD8^+^ T cells, HLA-Ib molecules are characterized by limited polymorphism and are thought to play specialized roles in immune regulation [64]. HLA-F has been reported to interact with several immune receptors expressed on natural killer (NK) cells and other innate immune populations, including members of the killer immunoglobulin-like receptor (KIR) family [38,65]. Through these interactions, HLA-F has been implicated in modulating NK cell activation, cytotoxicity, and cytokine production. Unlike HLA-G, whose expression in trophoblasts and role in maternal–fetal tolerance have been extensively studied [16,66], the expression pattern and functional relevance of HLA-F in the placenta remain poorly understood. Emerging evidence suggests that HLA-F expression can be modulated during viral infection, raising the possibility that it participates in the coordination of innate immune responses to pathogens [67]. This is particularly relevant at the maternal–fetal interface, where decidual NK cells represent the dominant immune population and play key roles in placental development and immune surveillance [68]. However, whether HLA-F may also function in soluble form to influence immune communication within the placental microenvironment remains largely unexplored.

Type I interferons are central mediators of the antiviral response and play an important role in shaping immune defenses at the maternal–fetal interface [30,69]. Trophoblasts are equipped with robust innate immune sensing pathways that allow them to detect viral nucleic acids and rapidly induce interferon signaling, leading to the activation of interferon-stimulated genes that restrict viral replication and coordinate immune responses. Our findings identify HLA-F as a previously unrecognized component of this interferon-responsive network in trophoblasts. Stimulation with poly(I:C), a synthetic analog of viral double-stranded RNA, resulted in the upregulation of HLA-F expression and the release of soluble HLA-F, suggesting that viral sensing pathways can directly regulate this non-classical HLA molecule. In contrast, infection with HSV-2, a virus known to interfere with type I interferon signaling [28,53], resulted in suppression of HLA-F expression, further supporting the idea that HLA-F is tightly linked to interferon-dependent antiviral responses.

An additional intriguing finding from our study is the detection of secreted HLA-F following poly(I:C) stimulation, suggesting that HLA-F may function not only as a cell-associated molecule but also as a soluble mediator within the placental microenvironment. Soluble forms of non-classical HLA molecules, particularly HLA-G, have been well documented and are known to exert potent immunomodulatory effects by regulating NK cell activity, T cell responses, and antigen-presenting cell function [70,71,72,73]. In this context, the presence of soluble HLA-F raises the possibility that it may participate in intercellular communication at the maternal–fetal interface, where precise regulation of immune activation is required to balance antiviral defense with fetal tolerance. The release of HLA-F in response to viral sensing pathways suggests that trophoblasts may use this molecule as part of a broader mechanism to modulate local immune responses during infection. Although the mechanisms underlying HLA-F secretion remain unclear, it is possible that non-classical trafficking pathways or stress-induced release contribute to its extracellular presence. To our knowledge, evidence for soluble HLA-F production by trophoblasts has been limited, making this observation particularly relevant for understanding immune communication at the maternal–fetal interface. Notably, the predominantly intracellular localization of HLA-F observed in trophoblasts further supports the possibility that its trafficking and release may follow non-classical pathways, an aspect that warrants further investigation.

A severe viral infection that can cross the placental barrier and disrupt fetal development is ZIKV [74,75]. Its ability to cause congenital abnormalities, including microcephaly, and pregnancy loss [24,76], has made it a major public health concern, particularly in pregnancy. ZIKV has been classified among the TORCH pathogens (Toxoplasma, Other, Rubella, Cytomegalovirus, and Herpes simplex virus), which are known to cause serious fetal morbidity and mortality [77].

The role of type I IFN responses in the anti-viral response has been shown in animal models [78], and trophoblasts appear capable of mounting a robust IFN-β response [31,43]. The critical role of this pathway is also demonstrated in IFNAR1^−^/^−^ mice, which show impaired antiviral defense and heightened susceptibility to ZIKV infection [79,80]. In this study we show that ZIKV infection in Sw71 trophoblasts led to increased HLA-F expression at both mRNA and protein levels, suggesting its role in the innate immune response against ZIKV infection.

Recent single-cell transcriptomic studies have reported reduced expression of HLA-F in placental trophoblast cells associated with preeclampsia [40], suggesting a potential link between HLA-F dysregulation and impaired immune tolerance at the maternal–fetal interface. Given that HLA-F can interact with receptors expressed on NK cells and other innate immune populations, alterations in HLA-F expression or secretion could influence the balance between immune tolerance and activation within the decidua. Such changes may contribute to the placental dysfunction and inflammatory environment characteristic of preeclampsia. Further studies examining HLA-F expression and function in placentas from preeclamptic pregnancies will be important to determine whether this molecule plays a role in the pathogenesis of this disorder.

In summary, our study identifies HLA-F as a previously unrecognized interferon-induced molecule in trophoblasts, linking viral sensing pathways to immune regulation at the maternal–fetal interface. We demonstrate that HLA-F expression in trophoblast cells is strongly influenced by IFNb, is induced following activation of antiviral pathways, and can be released in soluble form into the extracellular environment. The suppression of HLA-F expression during HSV-2 infection further supports the notion that viruses capable of interfering with interferon signaling may disrupt this regulatory pathway. Together, these findings suggest that HLA-F may contribute to the coordination of antiviral defense and immune modulation within the placental microenvironment. Given the critical importance of maintaining immune balance during early pregnancy, dysregulation of pathways controlling HLA-F expression could have implications for infection-associated pregnancy complications. Future studies will be necessary to define the immune receptors and cellular targets of trophoblast-derived HLA-F and to determine how this pathway influences immune cell function and pregnancy outcomes during viral infection. In addition, CRISPR-mediated manipulation of HLA-F in trophoblast cells will help determine whether HLA-F impacts viral replication in trophoblast cells.

## Data Availability

The data that support the findings of this study are available from the corresponding author upon reasonable request.

## Abbreviations

The following abbreviations are used in this manuscript:

CM	conditioned medium
dsRNA	double-stranded RNA
EVTs	extravillous trophoblasts
HLA	human leukocyte antigen
HLA-F	human leukocyte antigen F
HSV-2	herpes simplex virus type 2
IFN-β	interferon-β
ISGs	interferon-stimulated genes
KIRs	killer immunoglobulin-like receptors
MFI	maternal–fetal interface
NK	natural killer
PE	preeclampsia
Poly I:C	polyinosinic-polycytidylic acid
Sw71	Swan71
sHLA-F	soluble HLA-F
IFN	type-I interferon
ZIKV	Zika virus

## References

[1] Mor G., Cardenas I. (2010). The immune system in pregnancy: A unique complexity. Am. J. Reprod. Immunol..

[2] Mor G., Aldo P., Alvero A.B. (2017). The unique immunological and microbial aspects of pregnancy. Nat. Rev. Immunol..

[3] Norwitz E., Schust D., Fisher S. (2001). Implantation and the survival of early pregnancy. N. Engl. J. Med..

[4] Mor G., Cardenas I., Abrahams V., Guller S. (2011). Inflammation and pregnancy: The role of the immune system at the implantation site. Ann. N. Y. Acad. Sci..

[5] You Y., Stelzl P., Joseph D.N., Aldo P.B., Maxwell A.J., Dekel N., Liao A., Whirledge S., Mor G. (2021). TNF-α Regulated Endometrial Stroma Secretome Promotes Trophoblast Invasion. Front. Immunol..

[6] Sharkey A.M., Macklon N.S. (2013). The science of implantation emerges blinking into the light. Reprod. Biomed. Online.

[7] Pijnenborg R., Bland J.M., Robertson W.B., Brosens I. (1983). Uteroplacental arterial changes related to interstitial trophoblast migration in early human pregnancy. Placenta.

[8] Moser G., Weiss G., Sundl M., Gauster M., Siwetz M., Lang-Olip I., Huppertz B. (2017). Extravillous trophoblasts invade more than uterine arteries: Evidence for the invasion of uterine veins. Histochem. Cell Biol..

[9] Moser G., Gauster M., Orendi K., Glasner A., Theuerkauf R., Huppertz B. (2010). Endoglandular trophoblast, an alternative route of trophoblast invasion? Analysis with novel confrontation co-culture models. Hum. Reprod..

[10] Guzman-Genuino R.M., Dimova T., You Y., Aldo P., Hayball J.D., Mor G., Diener K.R. (2019). Trophoblasts promote induction of a regulatory phenotype in B cells that can protect against detrimental T cell-mediated inflammation. Am. J. Reprod. Immunol..

[11] Bulmer J.N., Williams P.J., Lash G.E. (2010). Immune cells in the placental bed. Int. J. Dev. Biol..

[12] Helige C., Ahammer H., Moser G., Hammer A., Dohr G., Huppertz B., Sedlmayr P. (2014). Distribution of decidual natural killer cells and macrophages in the neighbourhood of the trophoblast invasion front: A quantitative evaluation. Hum. Reprod..

[13] Trundley A., Moffett A. (2004). Human uterine leukocytes and pregnancy. Tissue Antigens.

[14] Ferreira L.M.R., Meissner T.B., Tilburgs T., Strominger J.L. (2017). HLA-G: At the Interface of Maternal-Fetal Tolerance. Trends Immunol..

[15] Lunemann S., Schobel A., Kah J., Fittje P., Holzemer A., Langeneckert A.E., Hess L.U., Poch T., Martrus G., Garcia-Beltran W.F. (2018). Interactions Between KIR3DS1 and HLA-F Activate Natural Killer Cells to Control HCV Replication in Cell Culture. Gastroenterology.

[16] Apps R., Murphy S.P., Fernando R., Gardner L., Ahad T., Moffett A. (2009). Human leucocyte antigen (HLA) expression of primary trophoblast cells and placental cell lines, determined using single antigen beads to characterize allotype specificities of anti-HLA antibodies. Immunology.

[17] Jamieson D.J., Theiler R.N., Rasmussen S.A. (2006). Emerging infections and pregnancy. Emerg. Infect. Dis..

[18] Pomar L., Musso D., Malinger G., Vouga M., Panchaud A., Baud D. (2019). Zika virus during pregnancy: From maternal exposure to congenital Zika virus syndrome. Prenat. Diagn..

[19] Patton M.E., Bernstein K., Liu G., Zaidi A., Markowitz L.E. (2018). Seroprevalence of Herpes Simplex Virus Types 1 and 2 Among Pregnant Women and Sexually Active, Nonpregnant Women in the United States. Clin. Infect. Dis..

[20] Brown Z.A., Selke S., Zeh J., Kopelman J., Maslow A., Ashley R.L., Watts D.H., Berry S., Herd M., Corey L. (1997). The acquisition of herpes simplex virus during pregnancy. N. Engl. J. Med..

[21] Dick G.W., Kitchen S.F., Haddow A.J. (1952). Zika virus. I. Isolations and serological specificity. Trans. R. Soc. Trop. Med. Hyg..

[22] Sadovsky Y., Clifton V.L., Knofler M. (2016). Editorial: ZIKA virus and placenta. Placenta.

[23] Shan C., Xie X., Muruato A.E., Rossi S.L., Roundy C.M., Azar S.R., Yang Y., Tesh R.B., Bourne N., Barrett A.D. (2016). An Infectious cDNA Clone of Zika Virus to Study Viral Virulence, Mosquito Transmission, and Antiviral Inhibitors. Cell Host Microbe.

[24] Schwartz D.A. (2017). The Origins and Emergence of Zika Virus, the Newest TORCH Infection: What’s Old Is New Again. Arch. Pathol. Lab. Med..

[25] Aliota M.T., Caine E.A., Walker E.C., Larkin K.E., Camacho E., Osorio J.E. (2016). Characterization of Lethal Zika Virus Infection in AG129 Mice. PLoS Negl. Trop. Dis..

[26] Lazear H.M., Diamond M.S. (2016). Zika Virus: New Clinical Syndromes and Its Emergence in the Western Hemisphere. J. Virol..

[27] Yockey L.J., Varela L., Rakib T., Khoury-Hanold W., Fink S.L., Stutz B., Szigeti-Buck K., Van den Pol A., Lindenbach B.D., Horvath T.L. (2016). Vaginal Exposure to Zika Virus during Pregnancy Leads to Fetal Brain Infection. Cell.

[28] Racicot K., Kwon J.Y., Aldo P., Abrahams V., El-Guindy A., Romero R., Mor G. (2016). Type I Interferon Regulates the Placental Inflammatory Response to Bacteria and is Targeted by Virus: Mechanism of Polymicrobial Infection-Induced Preterm Birth. Am. J. Reprod. Immunol..

[29] Kadeppagari R.K., Sanchez R.L., Foster T.P. (2012). HSV-2 inhibits type-I interferon signaling via multiple complementary and compensatory STAT2-associated mechanisms. Virus Res..

[30] Kwon J.Y., Aldo P., You Y., Ding J., Racicot K., Dong X., Murphy J., Glukshtad G., Silasi M., Peng J. (2018). Relevance of placental type I interferon beta regulation for pregnancy success. Cell. Mol. Immunol..

[31] You Y., Grasso E., Alvero A., Condon J., Dimova T., Hu A., Ding J., Alexandrova M., Manchorova D., Dimitrova V. (2023). Twist1-IRF9 Interaction Is Necessary for IFN-Stimulated Gene Anti-Zika Viral Infection. J. Immunol..

[32] Aldo P.B., Mulla M.J., Romero R., Mor G., Abrahams V.M. (2010). Viral ssRNA induces first trimester trophoblast apoptosis through an inflammatory mechanism. Am. J. Reprod. Immunol..

[33] Ding J., Aldo P., Roberts C.M., Stabach P., Liu H., You Y., Qiu X., Jeong J., Maxwell A., Lindenbach B. (2021). Placenta-derived interferon-stimulated gene 20 controls ZIKA virus infection. EMBO Rep..

[34] Atay S., Gercel-Taylor C., Suttles J., Mor G., Taylor D.D. (2011). Trophoblast-derived exosomes mediate monocyte recruitment and differentiation. Am. J. Reprod. Immunol..

[35] Ouyang Y., Mouillet J.F., Coyne C.B., Sadovsky Y. (2014). Review: Placenta-specific microRNAs in exosomes-good things come in nano-packages. Placenta.

[36] Goodridge J.P., Burian A., Lee N., Geraghty D.E. (2013). HLA-F and MHC class I open conformers are ligands for NK cell Ig-like receptors. J. Immunol..

[37] Lepin E.J., Bastin J.M., Allan D.S., Roncador G., Braud V.M., Mason D.Y., van der Merwe P.A., McMichael A.J., Bell J.I., Powis S.H. (2000). Functional characterization of HLA-F and binding of HLA-F tetramers to ILT2 and ILT4 receptors. Eur. J. Immunol..

[38] Dulberger C.L., McMurtrey C.P., Holzemer A., Neu K.E., Liu V., Steinbach A.M., Garcia-Beltran W.F., Sulak M., Jabri B., Lynch V.J. (2017). Human Leukocyte Antigen F Presents Peptides and Regulates Immunity through Interactions with NK Cell Receptors. Immunity.

[39] Sim M.J.W., Sun P.D. (2017). HLA-F: A New Kid Licensed for Peptide Presentation. Immunity.

[40] Luo F., Liu F., Guo Y., Xu W., Li Y., Yi J., Fournier T., Degrelle S., Zitouni H., Hernandez I. (2023). Single-cell profiling reveals immune disturbances landscape and HLA-F-mediated immune tolerance at the maternal-fetal interface in preeclampsia. Front. Immunol..

[41] Dunk C.E., Bucher M., Zhang J., Hayder H., Geraghty D.E., Lye S.J., Myatt L., Hackmon R. (2022). Human leukocyte antigen HLA-C, HLA-G, HLA-F, and HLA-E placental profiles are altered in early severe preeclampsia and preterm birth with chorioamnionitis. Am. J. Obstet. Gynecol..

[42] Xu R., Huang Y., Xie W., Luo D., Mei J., Liu X., Liu F., Luo F. (2025). HLA-F regulates the proliferation of trophoblast via PKM2-dependent glycolysis in the pathogenesis of preeclampsia. Mol. Med..

[43] Aldo P., You Y., Szigeti K., Horvath T.L., Lindenbach B., Mor G. (2016). HSV-2 enhances ZIKV infection of the placenta and induces apoptosis in first-trimester trophoblast cells. Am. J. Reprod. Immunol..

[44] Livak K.J., Schmittgen T.D. (2001). Analysis of relative gene expression data using real-time quantitative PCR and the 2(-Delta Delta C(T)) Method. Methods.

[45] Lanciotti R.S., Kosoy O.L., Laven J.J., Velez J.O., Lambert A.J., Johnson A.J., Stanfield S.M., Duffy M.R. (2008). Genetic and serologic properties of Zika virus associated with an epidemic, Yap State, Micronesia, 2007. Emerg. Infect. Dis..

[46] Lee S.K., Kim J.Y., Lee M., Gilman-Sachs A., Kwak-Kim J. (2012). Th17 and regulatory T cells in women with recurrent pregnancy loss. Am. J. Reprod. Immunol..

[47] Straszewski-Chavez S.L., Abrahams V.M., Alvero A.B., Aldo P.B., Ma Y., Guller S., Romero R., Mor G. (2009). The isolation and characterization of a novel telomerase immortalized first trimester trophoblast cell line, Swan 71. Placenta.

[48] Loumagne L., Baudhuin J., Favier B., Montespan F., Carosella E.D., Rouas-Freiss N. (2014). In vivo evidence that secretion of HLA-G by immunogenic tumor cells allows their evasion from immunosurveillance. Int. J. Cancer.

[49] Koga K., Aldo P.B., Mor G. (2009). Toll-like receptors and pregnancy: Trophoblast as modulators of the immune response. J. Obstet. Gynaecol. Res..

[50] Abrahams V.M., Schaefer T.M., Fahey J.V., Visintin I., Wright J.A., Aldo P.B., Romero R., Wira C.R., Mor G. (2006). Expression and secretion of antiviral factors by trophoblast cells following stimulation by the TLR-3 agonist, Poly(I: C). Hum. Reprod..

[51] Dang J., Tiwari S.K., Lichinchi G., Qin Y., Patil V.S., Eroshkin A.M., Rana T.M. (2016). Zika Virus Depletes Neural Progenitors in Human Cerebral Organoids through Activation of the Innate Immune Receptor TLR3. Cell Stem Cell.

[52] Adibi J.J., Marques E.T., Cartus A., Beigi R.H. (2016). Teratogenic effects of the Zika virus and the role of the placenta. Lancet.

[53] Zhang M., Liu Y., Wang P., Guan X., He S., Luo S., Li C., Hu K., Jin W., Du T. (2015). HSV-2 immediate-early protein US1 inhibits IFN-beta production by suppressing association of IRF-3 with IFN-beta promoter. J. Immunol..

[54] Shi T.L., Huang L.J., Xiong Y.Q., Zhong Y.Y., Yang J.J., Fu T., Lei X.F., Chen Q. (2018). The risk of herpes simplex virus and human cytomegalovirus infection during pregnancy upon adverse pregnancy outcomes: A meta-analysis. J. Clin. Virol..

[55] Fa F., Laup L., Mandelbrot L., Sibiude J., Picone O. (2020). Fetal and neonatal abnormalities due to congenital herpes simplex virus infection: A literature review. Prenat. Diagn..

[56] Felker A.M., Nguyen P., Kaushic C. (2021). Primary HSV-2 Infection in Early Pregnancy Results in Transplacental Viral Transmission and Dose-Dependent Adverse Pregnancy Outcomes in a Novel Mouse Model. Viruses.

[57] Glasner A., Oiknine-Djian E., Weisblum Y., Diab M., Panet A., Wolf D.G., Mandelboim O. (2017). Zika Virus Escapes NK Cell Detection by Upregulating Major Histocompatibility Complex Class I Molecules. J. Virol..

[58] Hershkovitz O., Zilka A., Bar-Ilan A., Abutbul S., Davidson A., Mazzon M., Kummerer B.M., Monsoengo A., Jacobs M., Porgador A. (2008). Dengue virus replicon expressing the nonstructural proteins suffices to enhance membrane expression of HLA class I and inhibit lysis by human NK cells. J. Virol..

[59] Momburg F., Mullbacher A., Lobigs M. (2001). Modulation of transporter associated with antigen processing (TAP)-mediated peptide import into the endoplasmic reticulum by flavivirus infection. J. Virol..

[60] Lobigs M., Blanden R.V., Mullbacher A. (1996). Flavivirus-induced up-regulation of MHC class I antigens; implications for the induction of CD8+ T-cell-mediated autoimmunity. Immunol. Rev..

[61] Guan X., Zhang M., Fu M., Luo S., Hu Q. (2019). Herpes Simplex Virus Type 2 Immediate Early Protein ICP27 Inhibits IFN-β Production in Mucosal Epithelial Cells by Antagonizing IRF3 Activation. Front. Immunol..

[62] Yang Y., Wang W., Weng J., Li H., Ma Y., Liu L., Ma W. (2022). Advances in the study of HLA class Ib in maternal-fetal immune tolerance. Front. Immunol..

[63] Møller H.I., Christiansen C.M., Klok F.R., Pedersen N.H., Blauenfeldt T., Finne K.F., Nielsen H.S., Hviid T.V.F. (2025). Investigations of HLA-F and HLA-G 3′UTR Polymorphisms in Preeclampsia and Fetal Growth Restriction Indicate a Possible Role of HLA-F-HLA-G Haplotypes and Diplotypes. HLA.

[64] Laaribi A.B., Zidi I., Hannachi N., Ben Yahia H., Chaouch H., Bortolotti D., Zidi N., Letaief A., Yacoub S., Boudabous A. (2015). Association of an HLA-G 14-bp Insertion/Deletion polymorphism with high HBV replication in chronic hepatitis. J. Viral Hepat..

[65] Alippe Y., Hatterschide J., Coyne C.B., Diamond M.S. (2025). Innate immune responses to pathogens at the maternal–fetal interface. Nat. Rev. Immunol..

[66] Hackmon R., Pinnaduwage L., Zhang J., Lye S.J., Geraghty D.E., Dunk C.E. (2017). Definitive class I human leukocyte antigen expression in gestational placentation: HLA-F, HLA-E, HLA-C, and HLA-G in extravillous trophoblast invasion on placentation, pregnancy, and parturition. Am. J. Reprod. Immunol..

[67] Ishitani A., Sageshima N., Lee N., Dorofeeva N., Hatake K., Marquardt H., Geraghty D.E. (2003). Protein expression and peptide binding suggest unique and interacting functional roles for HLA-E, F, and G in maternal-placental immune recognition. J. Immunol..

[68] Zhang X., Wei H. (2021). Role of Decidual Natural Killer Cells in Human Pregnancy and Related Pregnancy Complications. Front. Immunol..

[69] Silasi M., Cardenas I., Kwon J.Y., Racicot K., Aldo P., Mor G. (2015). Viral infections during pregnancy. Am. J. Reprod. Immunol..

[70] Yang L., Semmes E.C., Ovies C., Megli C., Permar S., Gilner J.B., Coyne C.B. (2022). Innate immune signaling in trophoblast and decidua organoids defines differential antiviral defenses at the maternal-fetal interface. eLife.

[71] Woon E.V., Nikolaou D., MacLaran K., Norman-Taylor J., Bhagwat P., Cuff A.O., Johnson M.R., Male V. (2022). Uterine NK cells underexpress KIR2DL1/S1 and LILRB1 in reproductive failure. Front. Immunol..

[72] Weetman A.P. (1999). The immunology of pregnancy. Thyroid.

[73] Wang J., Zhao S.J., Wang L.L., Lin X.X., Mor G., Liao A.H. (2022). Leukocyte immunoglobulin-like receptor subfamily B: A novel immune checkpoint molecule at the maternal-fetal interface. J. Reprod. Immunol..

[74] Brett-Major D.M., Roth C.E. (2016). Zika virus, emergencies, uncertainty and vulnerable populations. J. R. Coll. Physicians Edinb..

[75] Brasil P., Pereira J.P., Raja Gabaglia C., Damasceno L., Wakimoto M., Ribeiro Nogueira R.M., Carvalho de Sequeira P., Machado Siqueira A., Abreu de Carvalho L.M., Cotrim da Cunha D. (2016). Zika Virus Infection in Pregnant Women in Rio de Janeiro-Preliminary Report. N. Engl. J. Med..

[76] Alvarado M.G., Schwartz D.A. (2017). Zika Virus Infection in Pregnancy, Microcephaly, and Maternal and Fetal Health: What We Think, What We Know, and What We Think We Know. Arch. Pathol. Lab. Med..

[77] Coyne C.B., Lazear H.M. (2016). Zika virus-reigniting the TORCH. Nat. Rev. Microbiol..

[78] Yockey L.J., Jurado K.A., Arora N., Millet A., Rakib T., Milano K.M., Hastings A.K., Fikrig E., Kong Y., Horvath T.L. (2018). Type I interferons instigate fetal demise after Zika virus infection. Sci. Immunol..

[79] Lazear H.M., Govero J., Smith A.M., Platt D.J., Fernandez E., Miner J.J., Diamond M.S. (2016). A Mouse Model of Zika Virus Pathogenesis. Cell Host Microbe.

[80] Miner J.J., Cao B., Govero J., Smith A.M., Fernandez E., Cabrera O.H., Garber C., Noll M., Klein R.S., Noguchi K.K. (2016). Zika Virus Infection during Pregnancy in Mice Causes Placental Damage and Fetal Demise. Cell.

